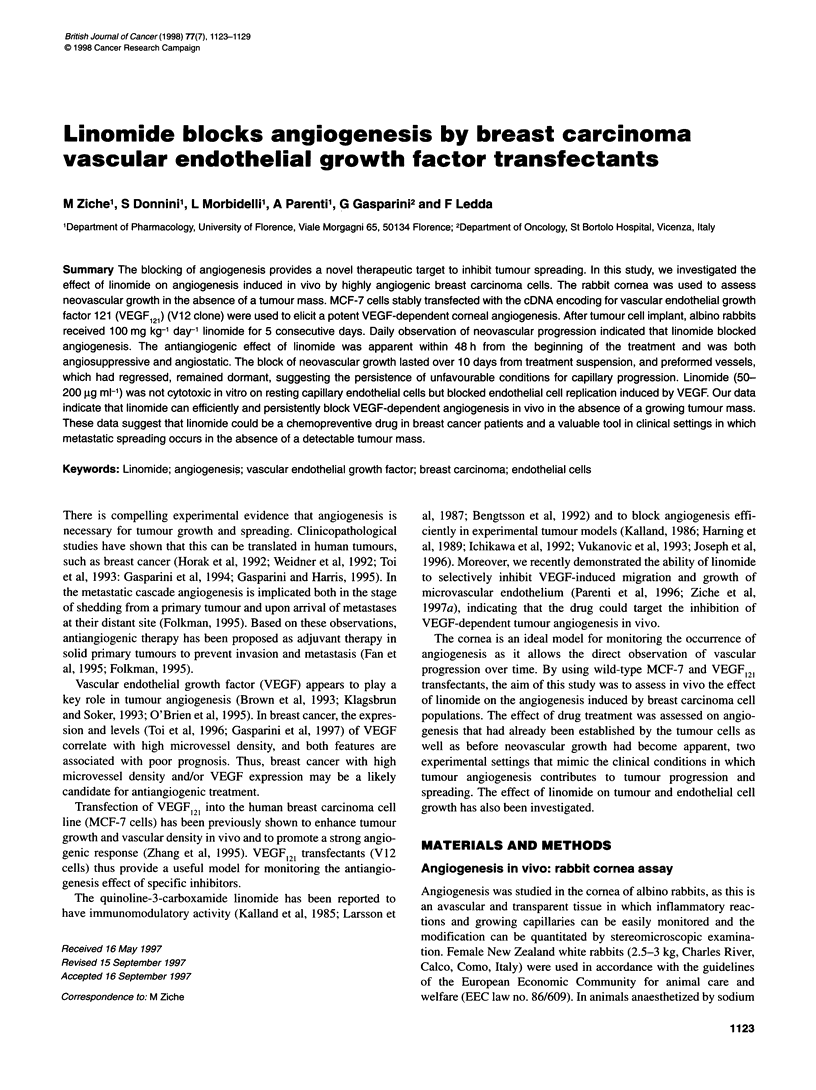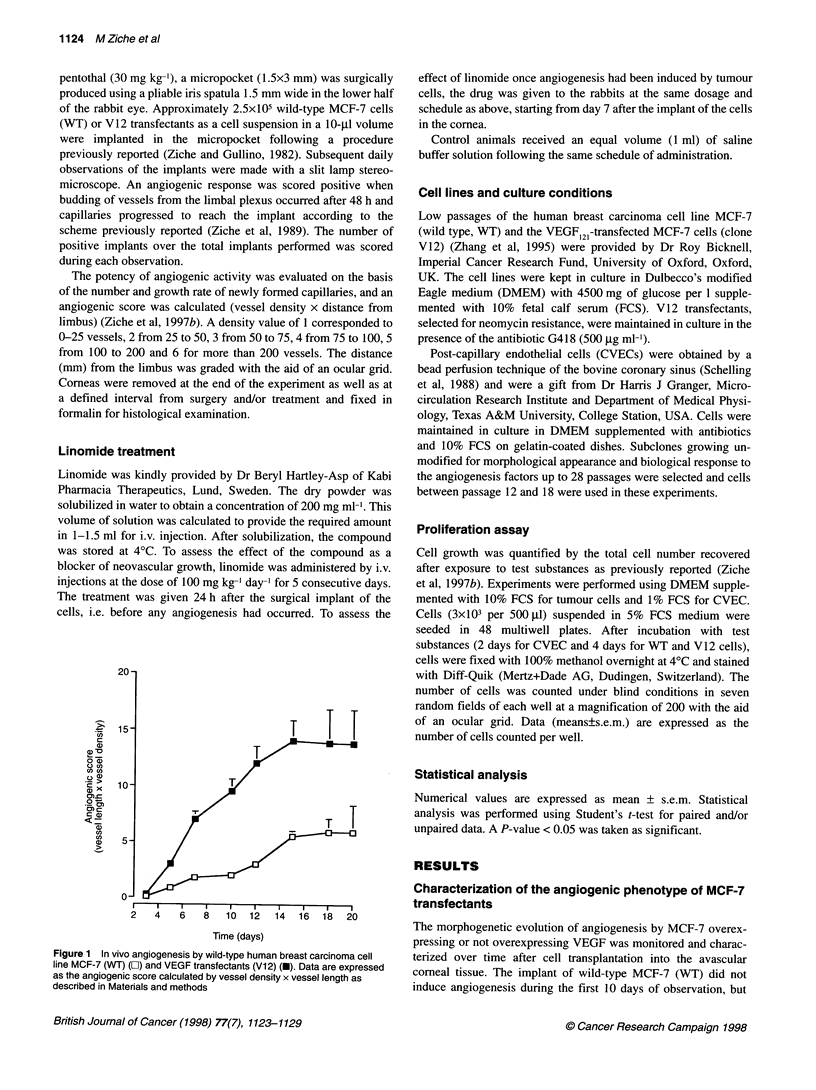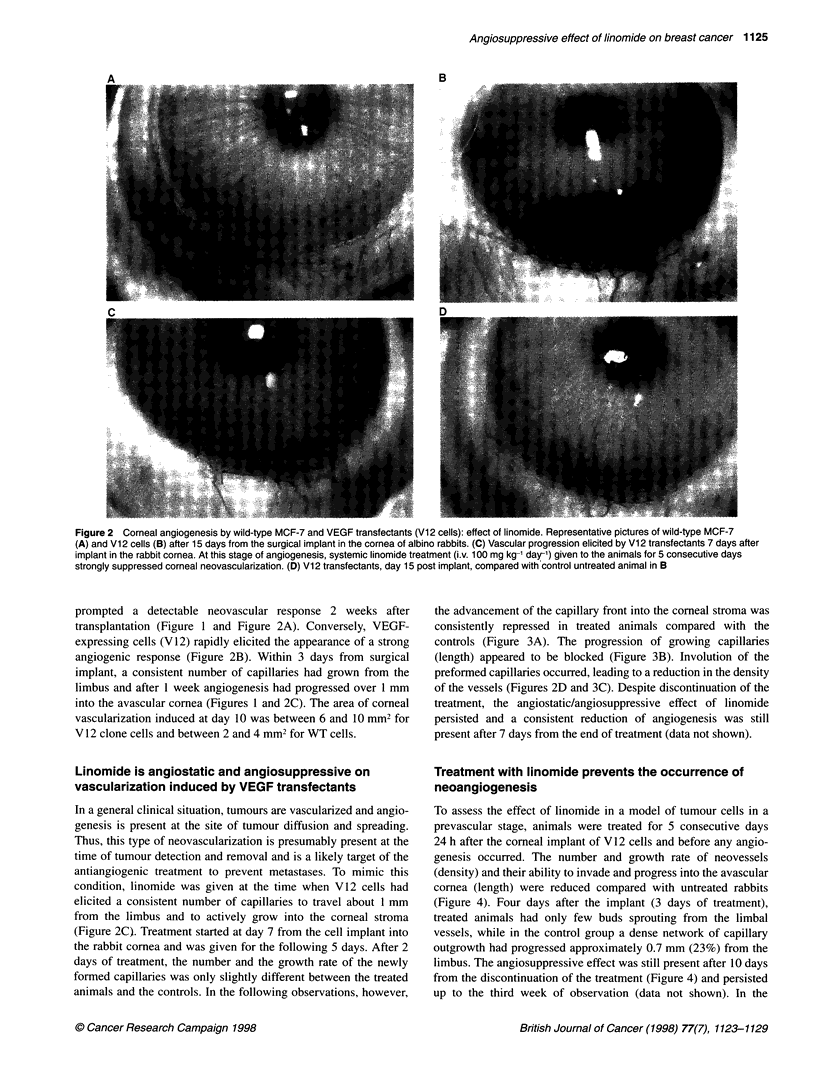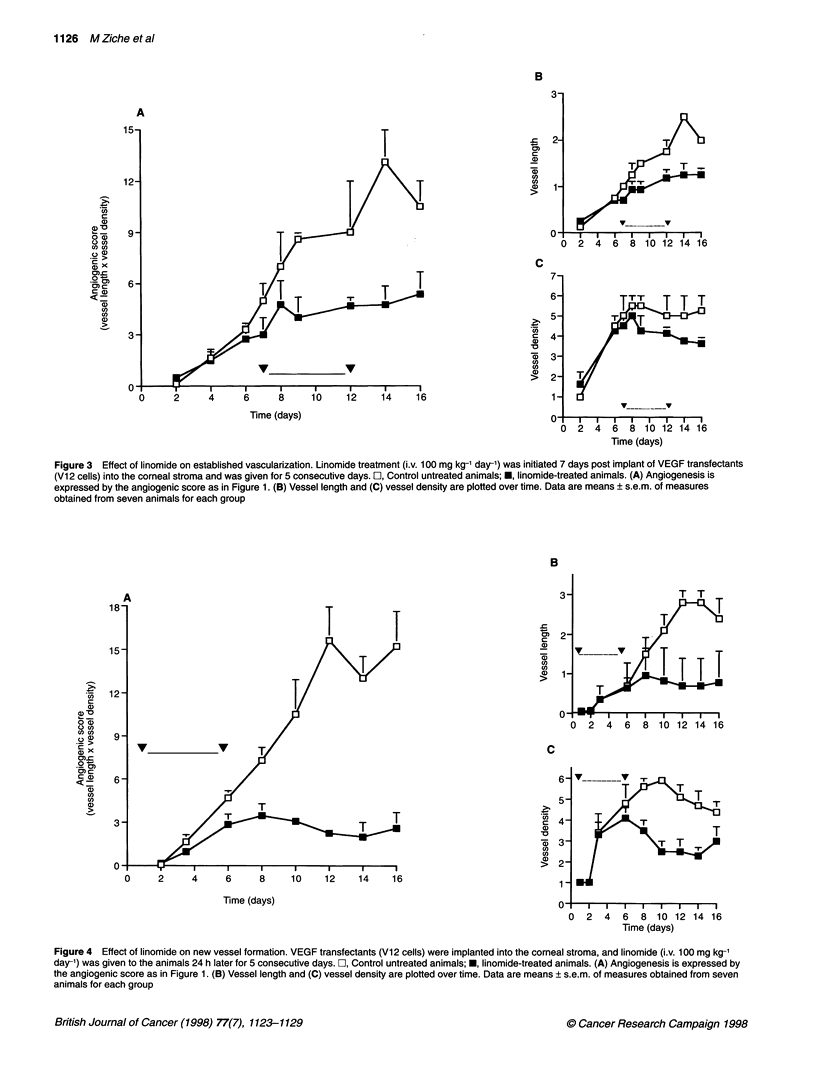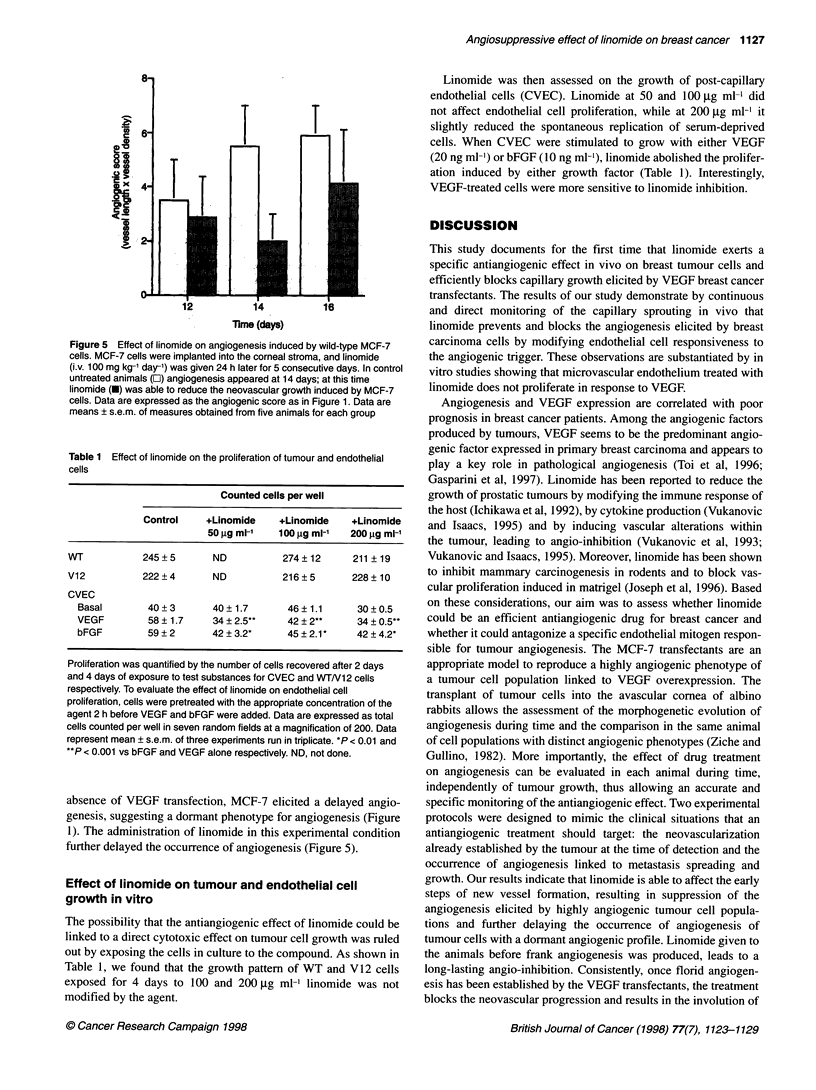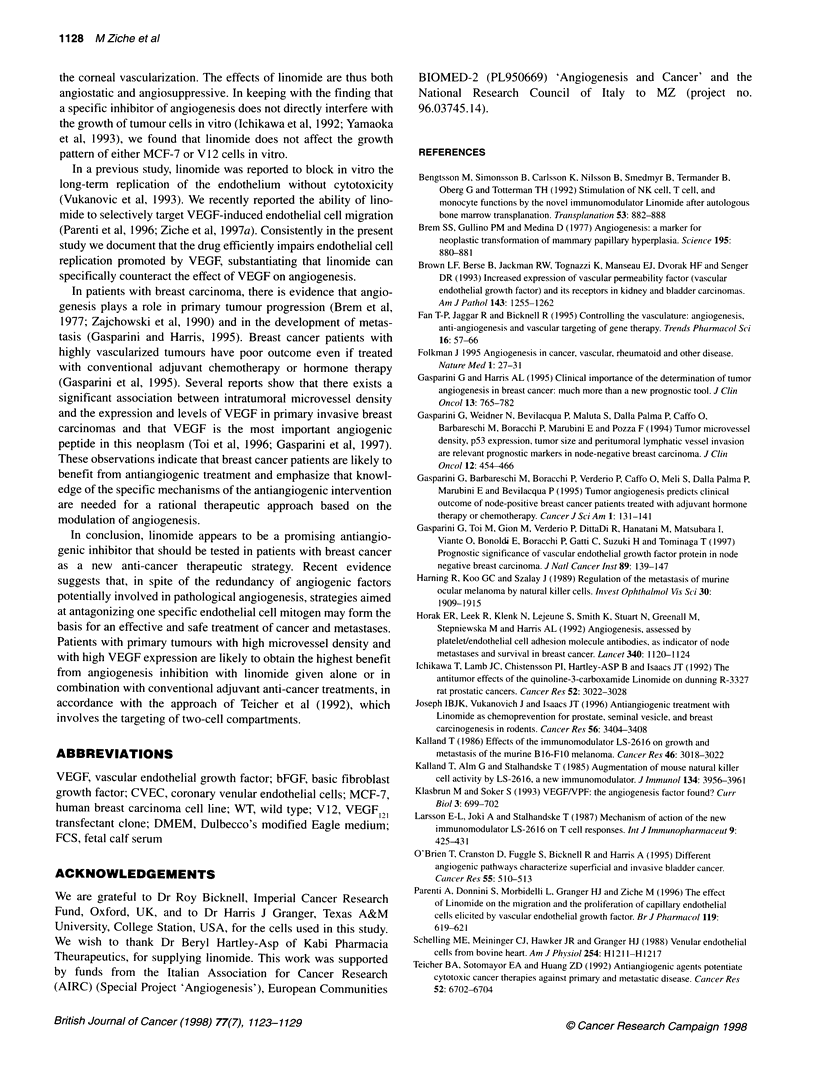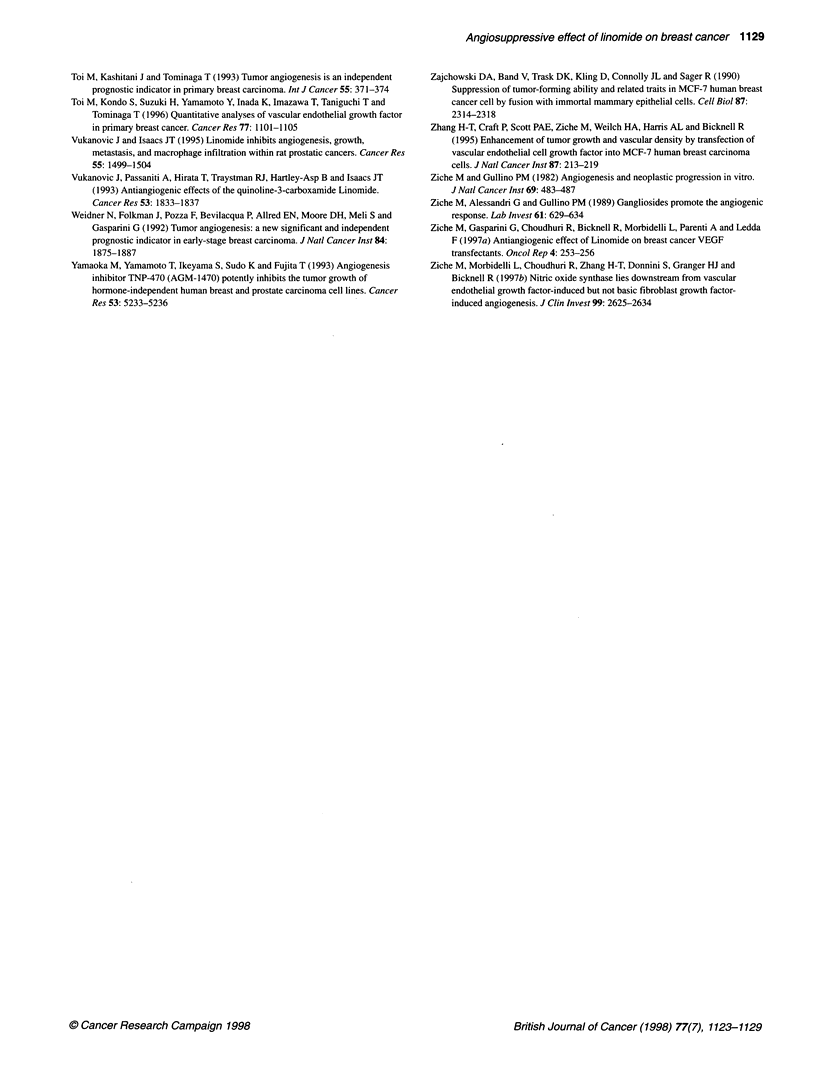# Linomide blocks angiogenesis by breast carcinoma vascular endothelial growth factor transfectants.

**DOI:** 10.1038/bjc.1998.186

**Published:** 1998-04

**Authors:** M. Ziche, S. Donnini, L. Morbidelli, A. Parenti, G. Gasparini, F. Ledda

**Affiliations:** Department of Pharmacology, University of Florence, Italy.

## Abstract

**Images:**


British Journal of Cancer (1998) 77(7), 1123–1129
© 1998 Cancer Research Campaign

### Linomide blocks angiogenesis by breast carcinoma vascular endothelial growth factor transfectants

**M Ziche[1], S Donnini[1], L Morbidelli[1], A Parenti[1], G Gasparini[2] and F Ledda**

[1]Department of Pharmacology, University of Florence, Viale Morgagni 65, 50134 Florence; [2]Department of Oncology, St Bortolo Hospital, Vicenza, Italy

**Summary** The blocking of angiogenesis provides a novel therapeutic target to inhibit tumour spreading. In this study, we investigated the effect of linomide on angiogenesis induced in vivo by highly angiogenic breast carcinoma cells. The rabbit cornea was used to assess neovascular growth in the absence of a tumour mass. MCF-7 cells stably transfected with the cDNA encoding for vascular endothelial growth factor 121 ($VEGF_{121}$) (V12 clone) were used to elicit a potent VEGF-dependent corneal angiogenesis. After tumour cell implant, albino rabbits received 100 mg $kg^{-1}$ $day^{-1}$ linomide for 5 consecutive days. Daily observation of neovascular progression indicated that linomide blocked angiogenesis. The antiangiogenic effect of linomide was apparent within 48 h from the beginning of the treatment and was both angiosuppressive and angiostatic. The block of neovascular growth lasted over 10 days from treatment suspension, and preformed vessels, which had regressed, remained dormant, suggesting the persistence of unfavourable conditions for capillary progression. Linomide (50–200 µg $ml^{-1}$) was not cytotoxic in vitro on resting capillary endothelial cells but blocked endothelial cell replication induced by VEGF. Our data indicate that linomide can efficiently and persistently block VEGF-dependent angiogenesis in vivo in the absence of a growing tumour mass. These data suggest that linomide could be a chemopreventive drug in breast cancer patients and a valuable tool in clinical settings in which metastatic spreading occurs in the absence of a detectable tumour mass.

**Keywords:** Linomide; angiogenesis; vascular endothelial growth factor; breast carcinoma; endothelial cells

There is compelling experimental evidence that angiogenesis is necessary for tumour growth and spreading. Clinicopathological studies have shown that this can be translated in human tumours, such as breast cancer (Horak et al, 1992; Weidner et al, 1992; Toi et al, 1993: Gasparini et al, 1994; Gasparini and Harris, 1995). In the metastatic cascade angiogenesis is implicated both in the stage of shedding from a primary tumour and upon arrival of metastases at their distant site (Folkman, 1995). Based on these observations, antiangiogenic therapy has been proposed as adjuvant therapy in solid primary tumours to prevent invasion and metastasis (Fan et al, 1995; Folkman, 1995).

Vascular endothelial growth factor (VEGF) appears to play a key role in tumour angiogenesis (Brown et al, 1993; Klagsbrun and Soker, 1993; O'Brien et al, 1995). In breast cancer, the expression and levels (Toi et al, 1996; Gasparini et al, 1997) of VEGF correlate with high microvessel density, and both features are associated with poor prognosis. Thus, breast cancer with high microvessel density and/or VEGF expression may be a likely candidate for antiangiogenic treatment.

Transfection of $VEGF_{121}$ into the human breast carcinoma cell line (MCF-7 cells) has been previously shown to enhance tumour growth and vascular density in vivo and to promote a strong angiogenic response (Zhang et al, 1995). $VEGF_{121}$ transfectants (V12 cells) thus provide a useful model for monitoring the antiangiogenesis effect of specific inhibitors.

The quinoline-3-carboxamide linomide has been reported to have immunomodulatory activity (Kalland et al, 1985; Larsson et al, 1987; Bengtsson et al, 1992) and to block angiogenesis efficiently in experimental tumour models (Kalland, 1986; Harning et al, 1989; Ichikawa et al, 1992; Vukanovic et al, 1993; Joseph et al, 1996). Moreover, we recently demonstrated the ability of linomide to selectively inhibit VEGF-induced migration and growth of microvascular endothelium (Parenti et al, 1996; Ziche et al, 1997*a*), indicating that the drug could target the inhibition of VEGF-dependent tumour angiogenesis in vivo.

The cornea is an ideal model for monitoring the occurrence of angiogenesis as it allows the direct observation of vascular progression over time. By using wild-type MCF-7 and $VEGF_{121}$ transfectants, the aim of this study was to assess in vivo the effect of linomide on the angiogenesis induced by breast carcinoma cell populations. The effect of drug treatment was assessed on angiogenesis that had already been established by the tumour cells as well as before neovascular growth had become apparent, two experimental settings that mimic the clinical conditions in which tumour angiogenesis contributes to tumour progression and spreading. The effect of linomide on tumour and endothelial cell growth has also been investigated.

*Received 16 May 1997*
*Revised 15 September 1997*
*Accepted 16 September 1997*

*Correspondence to:* M Ziche

### MATERIALS AND METHODS

### Angiogenesis in vivo: rabbit cornea assay

Angiogenesis was studied in the cornea of albino rabbits, as this is an avascular and transparent tissue in which inflammatory reactions and growing capillaries can be easily monitored and the modification can be quantitated by stereomicroscopic examination. Female New Zealand white rabbits (2.5–3 kg, Charles River, Calco, Como, Italy) were used in accordance with the guidelines of the European Economic Community for animal care and welfare (EEC law no. 86/609). In animals anaesthetized by sodium pentothal (30 mg kg$^{-1}$), a micropocket (1.5x3 mm) was surgically produced using a pliable iris spatula 1.5 mm wide in the lower half of the rabbit eye. Approximately 2.5x10$^5$ wild-type MCF-7 cells (WT) or V12 transfectants as a cell suspension in a 10-µl volume were implanted in the micropocket following a procedure previously reported (Ziche and Gullino, 1982). Subsequent daily observations of the implants were made with a slit lamp stereomicroscope. An angiogenic response was scored positive when budding of vessels from the limbal plexus occurred after 48 h and capillaries progressed to reach the implant according to the scheme previously reported (Ziche et al, 1989). The number of positive implants over the total implants performed was scored during each observation.

The potency of angiogenic activity was evaluated on the basis of the number and growth rate of newly formed capillaries, and an angiogenic score was calculated (vessel density × distance from limbus) (Ziche et al, 1997*b*). A density value of 1 corresponded to 0–25 vessels, 2 from 25 to 50, 3 from 50 to 75, 4 from 75 to 100, 5 from 100 to 200 and 6 for more than 200 vessels. The distance (mm) from the limbus was graded with the aid of an ocular grid. Corneas were removed at the end of the experiment as well as at a defined interval from surgery and/or treatment and fixed in formalin for histological examination.

### Linomide treatment

Linomide was kindly provided by Dr Beryl Hartley-Asp of Kabi Pharmacia Therapeutics, Lund, Sweden. The dry powder was solubilized in water to obtain a concentration of 200 mg ml$^{-1}$. This volume of solution was calculated to provide the required amount in 1–1.5 ml for i.v. injection. After solubilization, the compound was stored at 4°C. To assess the effect of the compound as a blocker of neovascular growth, linomide was administered by i.v. injections at the dose of 100 mg kg$^{-1}$ day$^{-1}$ for 5 consecutive days. The treatment was given 24 h after the surgical implant of the cells, i.e. before any angiogenesis had occurred. To assess the effect of linomide once angiogenesis had been induced by tumour cells, the drug was given to the rabbits at the same dosage and schedule as above, starting from day 7 after the implant of the cells in the cornea.

Control animals received an equal volume (1 ml) of saline buffer solution following the same schedule of administration.

**Figure 1** In vivo angiogenesis by wild-type human breast carcinoma cell line MCF-7 (WT) (□) and VEGF transfectants (V12) (■). Data are expressed as the angiogenic score calculated by vessel density × vessel length as described in Materials and methods

### Cell lines and culture conditions

Low passages of the human breast carcinoma cell line MCF-7 (wild type, WT) and the VEGF$_{121}$-transfected MCF-7 cells (clone V12) (Zhang et al, 1995) were provided by Dr Roy Bicknell, Imperial Cancer Research Fund, University of Oxford, Oxford, UK. The cell lines were kept in culture in Dulbecco's modified Eagle medium (DMEM) with 4500 mg of glucose per 1 supplemented with 10% fetal calf serum (FCS). V12 transfectants, selected for neomycin resistance, were maintained in culture in the presence of the antibiotic G418 (500 µg ml$^{-1}$).

Post-capillary endothelial cells (CVECs) were obtained by a bead perfusion technique of the bovine coronary sinus (Schelling et al, 1988) and were a gift from Dr Harris J Granger, Microcirculation Research Institute and Department of Medical Physiology, Texas A&M University, College Station, USA. Cells were maintained in culture in DMEM supplemented with antibiotics and 10% FCS on gelatin-coated dishes. Subclones growing unmodified for morphological appearance and biological response to the angiogenesis factors up to 28 passages were selected and cells between passage 12 and 18 were used in these experiments.

### Proliferation assay

Cell growth was quantified by the total cell number recovered after exposure to test substances as previously reported (Ziche et al, 1997*b*). Experiments were performed using DMEM supplemented with 10% FCS for tumour cells and 1% FCS for CVEC. Cells (3x10$^3$ per 500 µl) suspended in 5% FCS medium were seeded in 48 multiwell plates. After incubation with test substances (2 days for CVEC and 4 days for WT and V12 cells), cells were fixed with 100% methanol overnight at 4°C and stained with Diff-Quik (Mertz+Dade AG, Dudingen, Switzerland). The number of cells was counted under blind conditions in seven random fields of each well at a magnification of 200 with the aid of an ocular grid. Data (means±s.e.m.) are expressed as the number of cells counted per well.

### Statistical analysis

Numerical values are expressed as mean ± s.e.m. Statistical analysis was performed using Student's *t*-test for paired and/or unpaired data. A *P*-value < 0.05 was taken as significant.

### RESULTS

### Characterization of the angiogenic phenotype of MCF-7 transfectants

The morphogenetic evolution of angiogenesis by MCF-7 overexpressing or not overexpressing VEGF was monitored and characterized over time after cell transplantation into the avascular corneal tissue. The implant of wild-type MCF-7 (WT) did not induce angiogenesis during the first 10 days of observation, but

© Cancer Research Campaign 1998

**Figure 2** Corneal angiogenesis by wild-type MCF-7 and VEGF transfectants (V12 cells): effect of linomide. Representative pictures of wild-type MCF-7 (**A**) and V12 cells (**B**) after 15 days from the surgical implant in the cornea of albino rabbits. (**C**) Vascular progression elicited by V12 transfectants 7 days after implant in the rabbit cornea. At this stage of angiogenesis, systemic linomide treatment (i.v. 100 mg $kg^{-1}$ $day^{-1}$) given to the animals for 5 consecutive days strongly suppressed corneal neovascularization. (**D**) V12 transfectants, day 15 post implant, compared with control untreated animal in **B**

prompted a detectable neovascular response 2 weeks after transplantation (Figure 1 and Figure 2A). Conversely, VEGF-expressing cells (V12) rapidly elicited the appearance of a strong angiogenic response (Figure 2B). Within 3 days from surgical implant, a consistent number of capillaries had grown from the limbus and after 1 week angiogenesis had progressed over 1 mm into the avascular cornea (Figures 1 and 2C). The area of corneal vascularization induced at day 10 was between 6 and 10 $mm^2$ for V12 clone cells and between 2 and 4 $mm^2$ for WT cells.

### Linomide is angiostatic and angiosuppressive on vascularization induced by VEGF transfectants

In a general clinical situation, tumours are vascularized and angiogenesis is present at the site of tumour diffusion and spreading. Thus, this type of neovascularization is presumably present at the time of tumour detection and removal and is a likely target of the antiangiogenic treatment to prevent metastases. To mimic this condition, linomide was given at the time when V12 cells had elicited a consistent number of capillaries to travel about 1 mm from the limbus and to actively grow into the corneal stroma (Figure 2C). Treatment started at day 7 from the cell implant into the rabbit cornea and was given for the following 5 days. After 2 days of treatment, the number and the growth rate of the newly formed capillaries was only slightly different between the treated animals and the controls. In the following observations, however, the advancement of the capillary front into the corneal stroma was consistently repressed in treated animals compared with the controls (Figure 3A). The progression of growing capillaries (length) appeared to be blocked (Figure 3B). Involution of the preformed capillaries occurred, leading to a reduction in the density of the vessels (Figures 2D and 3C). Despite discontinuation of the treatment, the angiostatic/angiosuppressive effect of linomide persisted and a consistent reduction of angiogenesis was still present after 7 days from the end of treatment (data not shown).

### Treatment with linomide prevents the occurrence of neoangiogenesis

To assess the effect of linomide in a model of tumour cells in a prevascular stage, animals were treated for 5 consecutive days 24 h after the corneal implant of V12 cells and before any angiogenesis occurred. The number and growth rate of neovessels (density) and their ability to invade and progress into the avascular cornea (length) were reduced compared with untreated rabbits (Figure 4). Four days after the implant (3 days of treatment), treated animals had only few buds sprouting from the limbal vessels, while in the control group a dense network of capillary outgrowth had progressed approximately 0.7 mm (23%) from the limbus. The angiosuppressive effect was still present after 10 days from the discontinuation of the treatment (Figure 4) and persisted up to the third week of observation (data not shown). In the

**Figure 3** Effect of linomide on established vascularization. Linomide treatment (i.v. 100 mg kg$^{-1}$ day$^{-1}$) was initiated 7 days post implant of VEGF transfectants (V12 cells) into the corneal stroma and was given for 5 consecutive days. □, Control untreated animals; ■, linomide-treated animals. (**A**) Angiogenesis is expressed by the angiogenic score as in Figure 1. (**B**) Vessel length and (**C**) vessel density are plotted over time. Data are means ± s.e.m. of measures obtained from seven animals for each group

**Figure 4** Effect of linomide on new vessel formation. VEGF transfectants (V12 cells) were implanted into the corneal stroma, and linomide (i.v. 100 mg kg$^{-1}$ day$^{-1}$) was given to the animals 24 h later for 5 consecutive days. □, Control untreated animals; ■, linomide-treated animals. (**A**) Angiogenesis is expressed by the angiogenic score as in Figure 1. (**B**) Vessel length and (**C**) vessel density are plotted over time. Data are means ± s.e.m. of measures obtained from seven animals for each group

© Cancer Research Campaign 1998

**Figure 5** Effect of linomide on angiogenesis induced by wild-type MCF-7 cells. MCF-7 cells were implanted into the corneal stroma, and linomide (i.v. 100 mg $kg^{-1}$ $day^{-1}$) was given 24 h later for 5 consecutive days. In control untreated animals (□) angiogenesis appeared at 14 days; at this time linomide (■) was able to reduce the neovascular growth induced by MCF-7 cells. Data are expressed as the angiogenic score as in Figure 1. Data are means ± s.e.m. of measures obtained from five animals for each group

**Table 1** Effect of linomide on the proliferation of tumour and endothelial cells

| | Counted cells per well | | | |
|---|---|---|---|---|
| | Control | +Linomide 50 μg $ml^{-1}$ | +Linomide 100 μg $ml^{-1}$ | +Linomide 200 μg $ml^{-1}$ |
| WT | 245 ± 5 | ND | 274 ± 12 | 211 ± 19 |
| V12 | 222 ± 4 | ND | 216 ± 5 | 228 ± 10 |
| CVEC | | | | |
| Basal | 40 ± 3 | 40 ± 1.7 | 46 ± 1.1 | 30 ± 0.5 |
| VEGF | 58 ± 1.7 | 34 ± 2.5** | 42 ± 2** | 34 ± 0.5** |
| bFGF | 59 ± 2 | 42 ± 3.2* | 45 ± 2.1* | 42 ± 4.2* |

Proliferation was quantified by the number of cells recovered after 2 days and 4 days of exposure to test substances for CVEC and WT/V12 cells respectively. To evaluate the effect of linomide on endothelial cell proliferation, cells were pretreated with the appropriate concentration of the agent 2 h before VEGF and bFGF were added. Data are expressed as total cells counted per well in seven random fields at a magnification of 200. Data represent mean ± s.e.m. of three experiments run in triplicate. *$P < 0.01$ and **$P < 0.001$ vs bFGF and VEGF alone respectively. ND, not done.

absence of VEGF transfection, MCF-7 elicited a delayed angiogenesis, suggesting a dormant phenotype for angiogenesis (Figure 1). The administration of linomide in this experimental condition further delayed the occurrence of angiogenesis (Figure 5).

### Effect of linomide on tumour and endothelial cell growth in vitro

The possibility that the antiangiogenic effect of linomide could be linked to a direct cytotoxic effect on tumour cell growth was ruled out by exposing the cells in culture to the compound. As shown in Table 1, we found that the growth pattern of WT and V12 cells exposed for 4 days to 100 and 200 μg $ml^{-1}$ linomide was not modified by the agent.

Linomide was then assessed on the growth of post-capillary endothelial cells (CVEC). Linomide at 50 and 100 μg $ml^{-1}$ did not affect endothelial cell proliferation, while at 200 μg $ml^{-1}$ it slightly reduced the spontaneous replication of serum-deprived cells. When CVEC were stimulated to grow with either VEGF (20 ng $ml^{-1}$) or bFGF (10 ng $ml^{-1}$), linomide abolished the proliferation induced by either growth factor (Table 1). Interestingly, VEGF-treated cells were more sensitive to linomide inhibition.

### DISCUSSION

This study documents for the first time that linomide exerts a specific antiangiogenic effect in vivo on breast tumour cells and efficiently blocks capillary growth elicited by VEGF breast cancer transfectants. The results of our study demonstrate by continuous and direct monitoring of the capillary sprouting in vivo that linomide prevents and blocks the angiogenesis elicited by breast carcinoma cells by modifying endothelial cell responsiveness to the angiogenic trigger. These observations are substantiated by in vitro studies showing that microvascular endothelium treated with linomide does not proliferate in response to VEGF.

Angiogenesis and VEGF expression are correlated with poor prognosis in breast cancer patients. Among the angiogenic factors produced by tumours, VEGF seems to be the predominant angiogenic factor expressed in primary breast carcinoma and appears to play a key role in pathological angiogenesis (Toi et al, 1996; Gasparini et al, 1997). Linomide has been reported to reduce the growth of prostatic tumours by modifying the immune response of the host (Ichikawa et al, 1992), by cytokine production (Vukanovic and Isaacs, 1995) and by inducing vascular alterations within the tumour, leading to angio-inhibition (Vukanovic et al, 1993; Vukanovic and Isaacs, 1995). Moreover, linomide has been shown to inhibit mammary carcinogenesis in rodents and to block vascular proliferation induced in matrigel (Joseph et al, 1996). Based on these considerations, our aim was to assess whether linomide could be an efficient antiangiogenic drug for breast cancer and whether it could antagonize a specific endothelial mitogen responsible for tumour angiogenesis. The MCF-7 transfectants are an appropriate model to reproduce a highly angiogenic phenotype of a tumour cell population linked to VEGF overexpression. The transplant of tumour cells into the avascular cornea of albino rabbits allows the assessment of the morphogenetic evolution of angiogenesis during time and the comparison in the same animal of cell populations with distinct angiogenic phenotypes (Ziche and Gullino, 1982). More importantly, the effect of drug treatment on angiogenesis can be evaluated in each animal during time, independently of tumour growth, thus allowing an accurate and specific monitoring of the antiangiogenic effect. Two experimental protocols were designed to mimic the clinical situations that an antiangiogenic treatment should target: the neovascularization already established by the tumour at the time of detection and the occurrence of angiogenesis linked to metastasis spreading and growth. Our results indicate that linomide is able to affect the early steps of new vessel formation, resulting in suppression of the angiogenesis elicited by highly angiogenic tumour cell populations and further delaying the occurrence of angiogenesis of tumour cells with a dormant angiogenic profile. Linomide given to the animals before frank angiogenesis was produced, leads to a long-lasting angio-inhibition. Consistently, once florid angiogenesis has been established by the VEGF transfectants, the treatment blocks the neovascular progression and results in the involution of

© Cancer Research Campaign 1998

the corneal vascularization. The effects of linomide are thus both angiostatic and angiosuppressive. In keeping with the finding that a specific inhibitor of angiogenesis does not directly interfere with the growth of tumour cells in vitro (Ichikawa et al, 1992; Yamaoka et al, 1993), we found that linomide does not affect the growth pattern of either MCF-7 or V12 cells in vitro.

In a previous study, linomide was reported to block in vitro the long-term replication of the endothelium without cytotoxicity (Vukanovic et al, 1993). We recently reported the ability of linomide to selectively target VEGF-induced endothelial cell migration (Parenti et al, 1996; Ziche et al, 1997*a*). Consistently in the present study we document that the drug efficiently impairs endothelial cell replication promoted by VEGF, substantiating that linomide can specifically counteract the effect of VEGF on angiogenesis.

In patients with breast carcinoma, there is evidence that angiogenesis plays a role in primary tumour progression (Brem et al, 1977; Zajchowski et al, 1990) and in the development of metastasis (Gasparini and Harris, 1995). Breast cancer patients with highly vascularized tumours have poor outcome even if treated with conventional adjuvant chemotherapy or hormone therapy (Gasparini et al, 1995). Several reports show that there exists a significant association between intratumoral microvessel density and the expression and levels of VEGF in primary invasive breast carcinomas and that VEGF is the most important angiogenic peptide in this neoplasm (Toi et al, 1996; Gasparini et al, 1997). These observations indicate that breast cancer patients are likely to benefit from antiangiogenic treatment and emphasize that knowledge of the specific mechanisms of the antiangiogenic intervention are needed for a rational therapeutic approach based on the modulation of angiogenesis.

In conclusion, linomide appears to be a promising antiangiogenic inhibitor that should be tested in patients with breast cancer as a new anti-cancer therapeutic strategy. Recent evidence suggests that, in spite of the redundancy of angiogenic factors potentially involved in pathological angiogenesis, strategies aimed at antagonizing one specific endothelial cell mitogen may form the basis for an effective and safe treatment of cancer and metastases. Patients with primary tumours with high microvessel density and with high VEGF expression are likely to obtain the highest benefit from angiogenesis inhibition with linomide given alone or in combination with conventional adjuvant anti-cancer treatments, in accordance with the approach of Teicher et al (1992), which involves the targeting of two-cell compartments.

### ABBREVIATIONS

VEGF, vascular endothelial growth factor; bFGF, basic fibroblast growth factor; CVEC, coronary venular endothelial cells; MCF-7, human breast carcinoma cell line; WT, wild type; V12, $VEGF_{121}$ transfectant clone; DMEM, Dulbecco's modified Eagle medium; FCS, fetal calf serum

### ACKNOWLEDGEMENTS

We are grateful to Dr Roy Bicknell, Imperial Cancer Research Fund, Oxford, UK, and to Dr Harris J Granger, Texas A&M University, College Station, USA, for the cells used in this study. We wish to thank Dr Beryl Hartley-Asp of Kabi Pharmacia Theurapeutics, for supplying linomide. This work was supported by funds from the Italian Association for Cancer Research (AIRC) (Special Project 'Angiogenesis'), European Communities BIOMED-2 (PL950669) 'Angiogenesis and Cancer' and the National Research Council of Italy to MZ (project no. 96.03745.14).

### REFERENCES

Bengtsson M, Simonsson B, Carlsson K, Nilsson B, Smedmyr B, Termander B, Oberg G and Totterman TH (1992) Stimulation of NK cell, T cell, and monocyte functions by the novel immunomodulator Linomide after autologous bone marrow transplanation. *Transplanation* **53**: 882–888

Brem SS, Gullino PM and Medina D (1977) Angiogenesis: a marker for neoplastic transformation of mammary papillary hyperplasia. *Science* **195**: 880–881

Brown LF, Berse B, Jackman RW, Tognazzi K, Manseau EJ, Dvorak HF and Senger DR (1993) Increased expression of vascular permeability factor (vascular endothelial growth factor) and its receptors in kidney and bladder carcinomas. *Am J Pathol* **143**: 1255–1262

Fan T-P, Jaggar R and Bicknell R (1995) Controlling the vasculature: angiogenesis, anti-angiogenesis and vascular targeting of gene therapy. *Trends Pharmacol Sci* **16**: 57–66

Folkman J 1995 Angiogenesis in cancer, vascular, rheumatoid and other disease. *Nature Med* **1**: 27–31

Gasparini G and Harris AL (1995) Clinical importance of the determination of tumor angiogenesis in breast cancer: much more than a new prognostic tool. *J Clin Oncol* **13**: 765–782

Gasparini G, Weidner N, Bevilacqua P, Maluta S, Dalla Palma P, Caffo O, Barbareschi M, Boracchi P, Marubini E and Pozza F (1994) Tumor microvessel density, p53 expression, tumor size and peritumoral lymphatic vessel invasion are relevant prognostic markers in node-negative breast carcinoma. *J Clin Oncol* **12**: 454–466

Gasparini G, Barbareschi M, Boracchi P, Verderio P, Caffo O, Meli S, Dalla Palma P, Marubini E and Bevilacqua P (1995) Tumor angiogenesis predicts clinical outcome of node-positive breast cancer patients treated with adjuvant hormone therapy or chemotherapy. *Cancer J Sci Am* **1**: 131–141

Gasparini G, Toi M, Gion M, Verderio P, DittaDi R, Hanatani M, Matsubara I, Viante O, Bonoldi E, Boracchi P, Gatti C, Suzuki H and Tominaga T (1997) Prognostic significance of vascular endothelial growth factor protein in node negative breast carcinoma. *J Natl Cancer Inst* **89**: 139–147

Harning R, Koo GC and Szalay J (1989) Regulation of the metastasis of murine ocular melanoma by natural killer cells. *Invest Ophthalmol Vis Sci* **30**: 1909–1915

Horak ER, Leek R, Klenk N, Lejeune S, Smith K, Stuart N, Greenall M, Stepniewska M and Harris AL (1992) Angiogenesis, assessed by platelet/endothelial cell adhesion molecule antibodies, as indicator of node metastases and survival in breast cancer. *Lancet* **340**: 1120–1124

Ichikawa T, Lamb JC, Chistensson PI, Hartley-ASP B and Isaacs JT (1992) The antitumor effects of the quinoline-3-carboxamide Linomide on dunning R-3327 rat prostatic cancers. *Cancer Res* **52**: 3022–3028

Joseph IBJK, Vukanovich J and Isaacs JT (1996) Antiangiogenic treatment with Linomide as chemoprevention for prostate, seminal vesicle, and breast carcinogenesis in rodents. *Cancer Res* **56**: 3404–3408

Kalland T (1986) Effects of the immunomodulator LS-2616 on growth and metastasis of the murine B16-F10 melanoma. *Cancer Res* **46**: 3018–3022

Kalland T, Alm G and Stalhandske T (1985) Augmentation of mouse natural killer cell activity by LS-2616, a new immunomodulator. *J Immunol* **134**: 3956–3961

Klasbrun M and Soker S (1993) VEGF/VPF: the angiogenesis factor found? *Curr Biol* **3**: 699–702

Larsson E-L, Joki A and Stalhandske T (1987) Mechanism of action of the new immunomodulator LS-2616 on T cell responses. *Int J Immunopharmaceut* **9**: 425–431

O'Brien T, Cranston D, Fuggle S, Bicknell R and Harris A (1995) Different angiogenic pathways characterize superficial and invasive bladder cancer. *Cancer Res* **55**: 510–513

Parenti A, Donnini S, Morbidelli L, Granger HJ and Ziche M (1996) The effect of Linomide on the migration and the proliferation of capillary endothelial cells elicited by vascular endothelial growth factor. *Br J Pharmacol* **119**: 619–621

Schelling ME, Meininger CJ, Hawker JR and Granger HJ (1988) Venular endothelial cells from bovine heart. *Am J Physiol* **254**: H1211–H1217

Teicher BA, Sotomayor EA and Huang ZD (1992) Antiangiogenic agents potentiate cytotoxic cancer therapies against primary and metastatic disease. *Cancer Res* **52**: 6702–6704

© Cancer Research Campaign 1998

Toi M, Kashitani J and Tominaga T (1993) Tumor angiogenesis is an independent prognostic indicator in primary breast carcinoma. *Int J Cancer* **55**: 371–374

Toi M, Kondo S, Suzuki H, Yamamoto Y, Inada K, Imazawa T, Taniguchi T and Tominaga T (1996) Quantitative analyses of vascular endothelial growth factor in primary breast cancer. *Cancer Res* **77**: 1101–1105

Vukanovic J and Isaacs JT (1995) Linomide inhibits angiogenesis, growth, metastasis, and macrophage infiltration within rat prostatic cancers. *Cancer Res* **55**: 1499–1504

Vukanovic J, Passaniti A, Hirata T, Traystman RJ, Hartley-Asp B and Isaacs JT (1993) Antiangiogenic effects of the quinoline-3-carboxamide Linomide. *Cancer Res* **53**: 1833–1837

Weidner N, Folkman J, Pozza F, Bevilacqua P, Allred EN, Moore DH, Meli S and Gasparini G (1992) Tumor angiogenesis: a new significant and independent prognostic indicator in early-stage breast carcinoma. *J Natl Cancer Inst* **84**: 1875–1887

Yamaoka M, Yamamoto T, Ikeyama S, Sudo K and Fujita T (1993) Angiogenesis inhibitor TNP-470 (AGM-1470) potently inhibits the tumor growth of hormone-independent human breast and prostate carcinoma cell lines. *Cancer Res* **53**: 5233–5236

Zajchowski DA, Band V, Trask DK, Kling D, Connolly JL and Sager R (1990) Suppression of tumor-forming ability and related traits in MCF-7 human breast cancer cell by fusion with immortal mammary epithelial cells. *Cell Biol* **87**: 2314–2318

Zhang H-T, Craft P, Scott PAE, Ziche M, Weilch HA, Harris AL and Bicknell R (1995) Enhancement of tumor growth and vascular density by transfection of vascular endothelial cell growth factor into MCF-7 human breast carcinoma cells. *J Natl Cancer Inst* **87**: 213–219

Ziche M and Gullino PM (1982) Angiogenesis and neoplastic progression in vitro. *J Natl Cancer Inst* **69**: 483–487

Ziche M, Alessandri G and Gullino PM (1989) Gangliosides promote the angiogenic response. *Lab Invest* **61**: 629–634

Ziche M, Gasparini G, Choudhuri R, Bicknell R, Morbidelli L, Parenti A and Ledda F (1997*a*) Antiangiogenic effect of Linomide on breast cancer VEGF transfectants. *Oncol Rep* **4**: 253–256

Ziche M, Morbidelli L, Choudhuri R, Zhang H-T, Donnini S, Granger HJ and Bicknell R (1997*b*) Nitric oxide synthase lies downstream from vascular endothelial growth factor-induced but not basic fibroblast growth factor-induced angiogenesis. *J Clin Invest* **99**: 2625–2634

© Cancer Research Campaign 1998